# Nanoscale ultrastructures increase the visual conspicuousness of signalling traits in obligate cleaner shrimps

**DOI:** 10.1242/jeb.248064

**Published:** 2024-08-29

**Authors:** Eleanor M. Caves, Alexander L. Davis, Sönke Johnsen

**Affiliations:** ^1^ Department of Ecology, Evolution, and Marine Biology, University of California, Santa Barbara, Santa Barbara, CA 93106, USA; ^2^ Department of Ecology, Evolution, and Organismal Biology, Brown University, Providence, RI 02912, USA; ^3^ Department of Biology, Duke University, Durham, NC 27708, USA

**Keywords:** Interspecific signalling, Isoxanthopterin, Mutualism, Visual ecology

## Abstract

Signal theory predicts organisms should evolve signals that are conspicuous to intended receivers in natural signalling environments. Cleaner shrimps remove ectoparasites from reef fish clients and many signal their intent to clean by whipping long, white antennae. As white is a reliably conspicuous colour in aquatic environments, we hypothesized that selection has acted to increase broad-spectrum antennal reflectance in cleaners. Using scanning electron microscopy, optical models and reflectance measurements, we found that the antennae in three obligate cleaner species from two families (Palaemonidae and Lysmatidae) had thick (∼6 µm) chitinous layers or densely packed high refractive index spheres (300–400 nm diameter), which models show increase reflectance (400–700 nm). Two facultative and non-cleaning species had no visible antennae ultrastructure beyond the chitinous exoskeleton. Antennae reflectance was significantly higher in obligate cleaners than in facultative and non-cleaning species. Our results suggest that some obligate cleaners may have evolved ultrastructures that increase the conspicuousness of their antennae as signals.

## Teaser

**Highlighted Article:** Cleaner shrimp – tropical crustaceans that remove parasites from reef fish – advertise their cleaning services by waving long, white antennae which contain specialized nanostructures that increase antennae conspicuousness.

## INTRODUCTION

Organisms use visual signals for many functions, including attracting mates, warding off predators and communicating with mutualistic partners (e.g. Hill, 1991; Maan and Cummings, 2012; Cheney et al., 2009). Often, visual signals are colourful and are created using pigments (e.g. carotenoid-based signals found in various taxa: Kodric-Brown, 1989; Weaver et al., 2018) or nanostructures, such as those mediating the iridescent colours of butterflies and birds (Kemp and Rutowski, 2007; Silberglied and Taylor, 1978). Other signals are achromatic (e.g. white or black), and much of the work on achromatic signals has focused on the role of melanin pigments and ultrastructure in dark brown or black colouration (e.g. Da Silva et al., 2013; Galvan and Alonso-Alvarez, 2017). White as a signal colour, however, is also found across taxa, although less attention has been given to its structural basis (but see Stavenga et al., 2004; Wilts et al., 2017; Lemcoff et al., 2023). Revealing the structural basis of white signals in animals can help us understand how selection has shaped this widespread signal type.

**Fig. 1.**
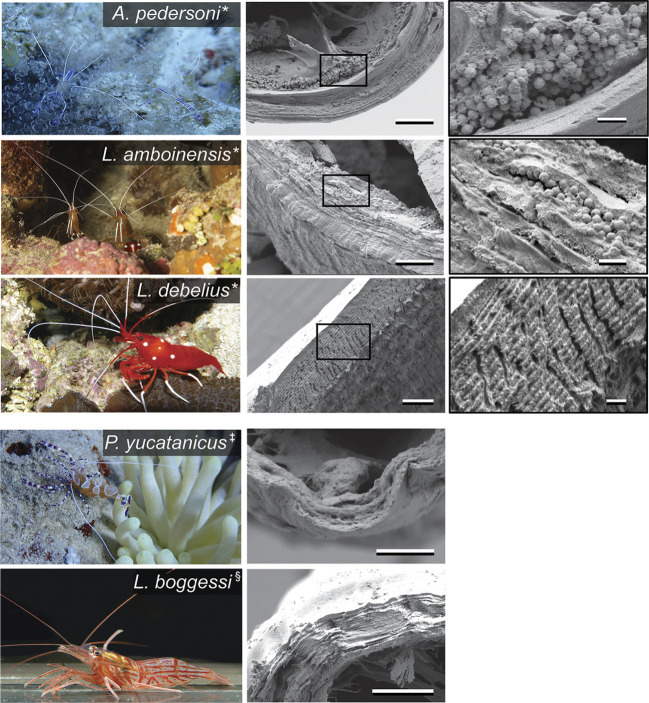
**Appearance (left) and antennae ultrastructure (middle and right) of the shrimp species.** We used three obligate cleaner species (*Ancylomenes pedersoni, *Lysmata amboinensis* and *Lysmata debelius*), one facultative cleaner (^‡^*Periclimenes yucatanicus*) and a related non-cleaner (^§^*Lysmata boggessi*). SEM images (middle) of the antennae show a sphere layer in *A. pedersoni* and *L. amboinensis*; densely packed chitin layers in *L. debelius*; and a chitin exoskeleton only in *P. yucatanicus* and *L. boggessi*. Scale bar: 5 µm. Higher magnification images (right) show structures that confer increased reflectance. Scale bars: 1 µm. Photo credits: Eleanor Caves (*A. pedersoni* and *P. yucatanicus*), Frank Gradyan (*L. amboinensis*), Haplochromis (*L. debelius*; CC BY-SA 3.0) and Chprieur (*L. boggessi*; CC BY-SA 4.0).

Cleaner shrimp are exclusively marine decapod crustaceans that remove and eat ectoparasites from dozens of species of coral reef fish clients (reviewed in Caves, 2021). Several species of cleaner shrimp from different families use white body parts to signal their intent to clean (Caves et al., 2018, 2019; Chapuis and Bshary, 2010; Wicksten, 2009). For example, *Ancylomenes pedersoni* (Palaemonidae) initiates cleaning interactions by whipping its white antennae (Caves et al., 2018), and several members of the genus *Lysmata* (Lysmatidae) advertise cleaning services by both rocking their white legs and waving their white antennae (Caves et al., 2019; Wicksten, 2009) (Fig. 1, left). White signals can be particularly effective when signalling to multiple species, as cleaner shrimp do, because they should appear conspicuous in most aquatic settings, regardless of the receiver's spectral sensitivity (colour vision capability) (Wilkins and Osorio, 2019). Furthermore, white signals, unlike coloured signals, should be effective across depths, water types and most viewing angles, as they create a reliably high achromatic contrast with most backgrounds. One challenge cleaner shrimp face, however, is that their antennae are only ∼150 µm in diameter, potentially limiting their detectability. One way to offset the limitation of these narrow diameters could be to evolve nanostructures that maximize reflectance from the antennae, similar to the pterin nanoparticles found on the wings of white pierid butterflies (Stavenga et al., 2004).

Here, we tested the hypothesis that cleaners are under selection to increase the visibility of their signals in a way that is effective for a range of visual systems across diverse visual environments, as is consistent with cleaners engaging with a large diversity of client fish types, across a range of depths and against a range of natural backgrounds. We used scanning electron microscopy (SEM), optical modelling and measurements of the reflectance of cleaner shrimp antennae to explore antennae ultrastructure and its effects on reflectance, in three obligate cleaner species in the families Palaemonidae (*Ancylomenes pedersoni*) and Lysmatidae (*Lysmata amboinensis*, *Lysmata debelius*) (Vaughan et al., 2017). For comparison, we conducted the same analyses in a species of facultative, or occasional, cleaner (Palaemonidae: *Periclimenes yucatanicus*; Titus et al., 2017) and one non-cleaner (Lysmatidae: *Lysmata boggessi*; Baeza, 2010) (Fig. 1).

## MATERIALS AND METHODS

### Specimens

Wild-caught specimens of the Caribbean *Ancylomenes pedersoni* (Chace 1958) (*n*=2), *Periclimenes yucatanicus* (Ives 1891) (*n*=3) and *Lysmata boggessi* Rhyne & Lin 2006 (*n*=3) were purchased from Dynasty Marine (Marathon, FL, USA), and wild-caught specimens of the Indo-Pacific *Lysmata amboinensis* (de Man 1888) (*n*=3) and *Lysmata debelius* Bruce 1983 (*n*=3), sourced from Indonesia, were purchased from LiveAquaria (Rhinelander, WI, USA). Shrimp were shipped within 1–2 days of being sourced from the wild, and within 6 h of arrival in the lab, were humanely euthanized by freezing, the antennae were removed, and the bodies were processed for DNA preservation for use in other projects. No formal ethical approvals were required for this work, as Decapod crustaceans are not currently covered under Institutional Animal Care and Use Committees in the USA.

### SEM

To identify underlying structural features that may enhance antennae reflectance, we used SEM. Two 3 mm long sections of antenna per animal were excised from two specimens per species and fixed for 12 h in 2.5% glutaraldehyde buffered with artificial seawater. After fixation, each sample went through a dehydration series of 30%, 30%, 50%, 50%, 70%, 70%, 90%, 90%, 100%, 100%, 100% ethanol (15 min per step). Samples were then dried using a LADD CPD3 critical point dryer (Ladd Research Industries, Williston, VT, USA) to preserve tissue ultrastructure. Once dried, samples were freeze-fractured to expose the cross-section, mounted on aluminium SEM stubs with copper tape, and sputter-coated with an ∼8 nm thick layer of gold (Denton Desk V, Denton Vacuum LLC, Moorestown, NJ, USA). The samples were imaged using an Apreo S scanning electron microscope (ThermoFisher Scientific, Waltham, MA, USA) at the Duke University Shared Materials and Instrumentation Facility with an acceleration voltage of 1 kV and magnifications of ×2500–15,000. We used Fiji (Schindelin et al., 2012) for morphometric analyses of the sphere layer found using SEM.

### Optical modelling

In two of the three obligate cleaner species, SEM images showed a layer of spherical nanoparticles inside the cuticle layer. To investigate how the morphology and optical properties of these particles impact antennae reflectance, we performed finite-difference time-domain simulations (FDTD) using the Lumerical solver version 2020b (Ansys, Canonsburg, PA, USA). Random close-packed aggregations of spheres mimicking the arrangement observed in the antennae were generated using the Uniform Random Particle Distribution (URPD) structure in Lumerical.

We performed three sets of simulations to determine the effects of sphere refractive index, sphere layer thickness and sphere layer diameter on reflectance. First, to determine the effect of sphere refractive index on reflectance, we simulated a 2 µm thick layer of non-absorbing spheres underneath a 5 µm layer of homogeneous chitin, varying sphere refractive index from 1.57 (approximately that of chitin) to 2.00 (a lower estimate of that of the pteridine granules found in white Pierid butterflies; Wilts et al., 2017) in increments of 0.01. Notably, this range encompassed the refractive index of isoxanthopterin (1.78), which prior work has identified as the molecular basis of the spheres underlying white body colouration in *L. amboinensis* (one of the obligate cleaners studied here) (Lemcoff et al., 2023). Second, we simulated layers of spheres (randomly assigned diameters between 300 and 400 nm, based on what was found in *A. pedersoni* and *L. amboinensis*) between 1 and 10 µm thick in 300 nm thickness intervals to determine how reflectance changes with layer thickness. Third, we simulated the effects of sphere diameter on the reflectance of a 2 µm thick layer of spheres (again based on what we observed in *A. pedersoni* and *L. amboinensis*) ranging between 100 and 1000 nm in diameter, in 30 nm intervals; within each simulation, all spheres had the same diameter (to within 5 nm of one another). For investigating the effects of both layer thickness and sphere diameter, we simulated three different sphere refractive indices, 1.57 (chitin), 1.78 (isoxanthopterin) and 2.00 (pteridine), with a background matrix equal to the refractive index of seawater (1.34).

All simulations were performed in a 4 µm×4 µm×12 µm domain with periodic boundary conditions in the *x* and *y* directions, broadband (400–700 nm) plane wave source propagating in the *z* direction, and perfectly matched layer boundary conditions in the *z* direction (a computational representation of open boundaries that does not reflect any light at the edges of the domain). Although we simulated one sphere layer, antennae are effectively one layer wrapped into a cylinder, meaning that most incident light upon the antennae passes through two layers. Therefore, we report reflectance as: *R*+*RT*^2^, where *R* is the reflectance of one layer and *T* is the transmittance, as this equation accounts for reflectance of the front layer, plus reflectance of the back layer after having passed through the front layer twice.

### Reflectance measurements

Based on the results of the SEM and optical modelling (above), we made predictions about relative antennal reflectance in each of our focal species. To quantify reflectance and test these predictions, we used calibrated photography under a stereomicroscope to measure reflectance from the antennae following methods in Tedore and Johnsen (2012). Calibrated photography was used instead of spectoradiometry because cleaner shrimp antennae are cylindrical rather than flat, causing light to scatter in directions that are not captured by the fibre optic sensor, probably underestimating reflectance. In brief, photographs of antennae were taken with an iPhone (model 13, Apple Inc., Cupertino, CA, USA) and included a reflectance standard comprising five grey paint swatches of measured reflectance. We measured the 0–255 value from each swatch of the grey standard [using the image analysis software Fiji (Schindelin et al., 2012)], using only the green channel of the sRGB image. The green channel was used both to approximate brightness vision in particular, as closely as we are able, and because, of the three colour channels, it is most closely aligned with the ambient illuminant on a coral reef (Cronin et al., 2014). We then plotted the green values of the grey standards against their known reflectance values, and fitted exponential equations to these values to create a calibration equation for each photograph. Then, in Fiji, we measured green-channel values from 4 to 10 regions on each antenna; the number of regions sampled did not affect our results, as randomly sampling only 4 regions from each specimen and re-running our analyses did not change our conclusions in any instance. We then converted these antennae values to reflectance using the calibration equations generated from the grey standards. We averaged reflectance values across individuals to yield a single measure of antennal reflectance for each specimen, and averaged across specimens to yield average values for each species, for use in analysis.

To determine whether cleaner ‘status’ predicts reflectance, we used two approaches. First, we classified species as either an ‘obligate’ or ‘non-obligate’ (which included both the facultative *P. yucatanicus* and the non-cleaner *L. boggessi*) cleaner and used an unpaired two-samples Wilcoxon test to determine whether mean reflectance differed significantly between these two groups. Second, we built a linear model using the *lm* function in R (http://www.R-project.org/) in which reflectance was the response variable and cleaner status (‘obligate’, ‘facultative’ or ‘non-cleaner’) was a fixed effect. We assessed the significance of the fixed effect by using an ANOVA to compare the fit of a full model, which included the fixed effect, with that of a model without the fixed effect using the ‘afex’ package (https://CRAN.R-project.org/package=afex).

We then investigated whether, among shrimps with white antennae, the presence of ultrastructures is associated with significantly higher antennal reflectance, using the same two methods as above. First, we compared antennal reflectance in the three obligate cleaner species with that of the facultative *P. yucatanicus* using an unpaired two-samples Wilcoxon test, and second, we used the same model as above but with species labelled as either ‘ultrastructures present’ (*A. pedersoni*, *L. amboinensis* and *L. debelius*) or ‘ultrastructures absent’ (*P. yucatanicus*). All analyses were performed in R version 4.0.3 (http://www.R-project.org/).

## RESULTS AND DISCUSSION

### Obligate cleaners have visible antennae ultrastructure

The facultative cleaner *P. yucatanicus* and the non-cleaner *L. boggessi* had no visible structures in their antennae that increased reflectance beyond a homogeneous chitinous exoskeleton. However, antennae in all three obligate cleaners contained visible structures. The antennae of both *A. pedersoni* and *L. amboinensis* contained a layer of spheres (Fig. 1). Spheres were slightly larger in *A. pedersoni* (diameter of 395±40 nm, mean±s.d., *n*=207 spheres from two specimens) than in *L. amboinensis* (363±42 nm, *n*=212 spheres from two specimens; Fig. S1). It was difficult to measure the exact thicknesses of the sphere layers because of distortions from freeze fracturing, but based on measurements in Fiji they appeared to be between 1 and 3 µm in both species. Antennae in the obligate cleaner *L. debelius* had extremely regular layers of chitin stacked tightly together in a structure approximately 6 µm thick.

**Fig. 2.**
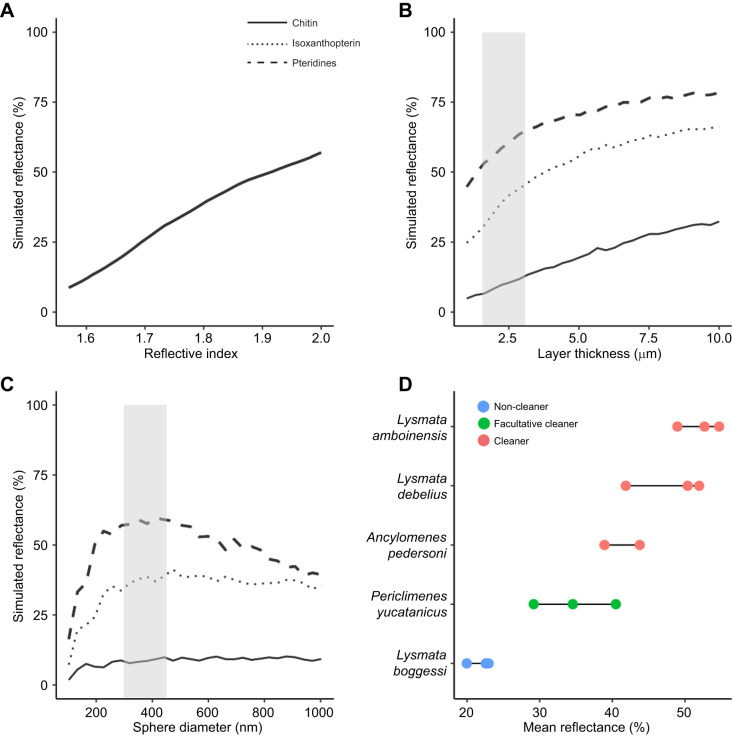
**Antennae reflectance and FDTD modelling of sphere layers.** (A) Simulated reflectance across the visible range (400–700 nm) from a 2 µm layer of spheres underneath a 5 µm chitin layer as refractive index is varied. (B,C) FDTD (finite-difference time-domain) simulations of (B) layers of varying thickness and (C) layers 2 µm thick but with varying sphere diameters, for refractive indices of chitin (1.57; solid line), isoxanthopterin (1.78; dotted line) and pteridines (2.00: dashed line). (D) Mean green-channel reflectance of antennae using calibrated colour photography in all five focal species [dots are individuals; antennae are white in *L. amboinensis* (*n*=3), *L. debelius* (*n*=3), *A. pedersoni* (*n*=2) and *P. yucatanicus* (*n*=3) and red in *L. boggessi* (*n*=3)]. In B and C, grey shaded regions represent ranges of layer thickness and sphere diameter, respectively, observed in the obligate cleaner shrimp *A. pedersoni* and *L. amboinensis*.

We first used FDTD modelling to assess the impact of the sphere layers found in *A. pedersoni* and *L. amboinensis* on reflectance and whether the sphere layers are optimal to increase reflectance. We found a strong, positive, linear effect of refractive index on reflectance, with the reflectance from a 2 µm layer increasing from 8.7% at a refractive index of 1.57 to 57% at a refractive index of 2.0 (Fig. 2A). Our control simulation, a 5 µm chitin layer with no sphere layer, had a reflectance of 1.9%. Even the addition of relatively low refractive index spheres (refractive index=1.57) to this chitin layer increased reflectance ∼4.6× compared with the chitin layer alone (1.9% versus 8.7%). With a refractive index of 1.78, spheres composed of isoxanthopterin increased reflectance over 19-fold (1.9% reflectance for a 5 µm chitin layer versus 36.9% reflectance for a chitin layer backed by a 2 µm layer of isoxanthopterin spheres).

The second parameter we varied in our simulations was sphere layer thickness. We performed 30 simulations of layers 1–10 µm thick – with observed layer thickness in cleaner shrimps lying at the lower end of this range, at approximately 2–3 μm thick – and found that increasing the layer thickness caused reflectance to increase (Fig. 2B). There was a strong linear relationship between thickness and reflectance for thin layers, with a decreasing slope as thickness approached 10 µm. The shape of the relationship between layer thickness and reflectance was refractive index dependent. The lower the refractive index, the thicker a layer must be to reach its asymptotic reflectance.

Third, we examined the effect of sphere diameter. Our modelling showed a sharp increase in reflectance at two of the refractive indices used as sphere diameter increased from 100 to 400 nm (Fig. 2C), and a plateau in reflectance for all three refractive indices, once sphere diameters reached 400 nm. For refractive indices of 1.78 and 1.57, reflectance plateaued at 38% and 10%, respectively, and for a refractive index of 2.00, reflectance plateaued at 60%. Thus, our simulations indicate that the size of spheres found in the antennae of *L. amboinensis* and *A. pedersoni* (Fig. 2C, grey shaded area) maximizes reflectance.

Notably, in the cleaner *L. debelius*, rather than a layer of spherical nanoparticles, we found highly regular stacks of chitin layers (Fig. 1) in addition to the ordered layering of the chitinous exoskeleton (such as that visible in *L. boggessi*). Each layer was approximately 0.27±0.06 µm (mean±s.d., based on measurements of *n*=10 layers) thick, stacked many layers high, for a total layer thickness of ∼6 µm. Although it is difficult to precisely model the optical implications of this structure based on the SEM images, stacks of sub-micrometre high index layers are typically associated with high reflectance over at least some portion of the visible spectrum (e.g. McKenzie et al., 1995).

### Antennae reflectance is higher in obligate cleaners

Based on the simulations described above, we predicted that the cleaners *A. pedersoni*, *L. amboinensis* and *L. debelius* would have higher reflectance than both the non-obligate cleaner *P. yucatanicus* and the non-cleaner *L. boggessi.* Reflectance measurements using calibrated colour photography upheld these predictions. Mean (±s.d.) reflectance in the green channel was higher in the three species of obligate cleaner (*A. pedersoni* 43.8% and 38.9%, *n*=2; *L. amboinensis* 52.1±4.0%, *n*=3; *L. debelius* 48.1±0.40%, *n*=3) than in either the facultative cleaner *P. yucatanicus* (34.7±1.6%, *n*=3) or the non-cleaner *L. boggessi* (21.8±2.3%, *n*=3) (Fig. 2D). Grouping the three obligate cleaners together and the two non-obligate cleaners together showed that reflectance was significantly higher in the obligate cleaners than in the non-obligate cleaners (unpaired two-samples Wilcoxon test, *P*=0.004), and a modelling approach showed that cleaner ‘status’ (‘obligate’, ‘facultative’ or ‘non-cleaner’) was a significant predictor of antennae reflectance (*P*<0.0001).

We also examined whether, among species with white antennae (*L. debelius*, *L. amboinensis*, *A. pedersoni* and *P. yucatanicus*), the presence of ultrastructure is associated with significantly higher reflectance. Comparison of reflectance showed it was significantly higher in the species with ultrastructure (*L. debelius*, *L. amboinensis* and *A. pedersoni*) than in the species without (*P. yucatanicus*) (unpaired two-samples Wilcoxon test, *P*=0.03). Similarly, a model in which reflectance was predicted by the presence or absence of ultrastructure in species with white antennae showed that the presence of ultrastructure is a significant predictor of reflectance (*P*=0.002).

Although the reflectance measured here was lower than other bright white colour patches in animals such as *Pieris rapae* wings (60–80%; Stavenga et al., 2004) or the white stripes of *Sepia officinalis* (60–70%; Mäthger et al., 2013), the reflectance measured here is likely an underestimate because of the cylindrical nature and small diameter of the tissue. Additionally, our calibrated colour photography setup did not allow us to measure reflectance in the ultraviolet, but this would be a particularly interesting future direction, given that ultraviolet-rich blues have been shown to be an important aspect of the ‘guild colouration’ of cleaner fish (Cheney et al., 2009).

### Conclusions

Our results show that three species of obligate cleaner shrimp that use their white antennae to signal to client fish, *A. pedersoni*, *L. amboinensis* and *L. debelius*, have structures that increase antennae reflectance. *Lysmata debelius* has a thick chitinous stack of high index layers, in addition to the chitinous exoskeleton also seen in the non-cleaners, that likely serves to increase reflectance, whereas *A. pedersoni* and *L. amboinensis*, despite being in different families, each have evolved 1–3 µm thick layers of spheres inside their antennae. Modelling suggests that these spheres are of a diameter that maximizes reflectance, and that this sphere layer increases reflectance by 4.6–19× compared with a 5 µm chitin layer with no spheres underneath. We assume here that these spheres are made of isoxanthopterin, a material found in reflective layers in other decapod crustaceans, including isoxanthopterin granules forming the bright white stripe down the body of *L. amboinensis* (Lemcoff et al., 2023) (a species also examined in this study), retinal reflectors of *Cherax quadricarinatus* (Palmer et al., 2018), *Homarus americanus* (Kleinholz, 1959), *Litopenaeus vannamei* (Palmer et al., 2020), *Machrobrachium rosenbergii* (Palmer et al., 2018) and *Penaeus setiferus* (Zyznar and Nicol, 1971), and reflectors in the photophores of the deep-sea shrimp *Oplophorus spinosus* and *Systellaspis debilis* (Nowel et al., 1998). Thus, cleaner shrimp may be using a widespread structural toolkit in a novel way to increase antennal reflectance.

By contrast, both the facultative cleaner *P. yucatanicus* and the non-cleaner *L. boggessi* appeared to have no structures in their antennae that increase reflectance. Interestingly, *P. yucatanicus* still has antennae that appear white, which likely occurs because their antennae have a large number of refractive index interfaces – both at the surface and internally – combined with low absorption, which scatters light across wavelengths and appears white. However, random close-packed spheres of a certain size scatter light over a broad wavelength and angle range very efficiently, in line with our finding that even though *P. yucatanicus* antennae appear white, they do not reflect as much light as the antennae of obligate cleaners that contain specialized nanostructures. Behaviourally, *P. yucatanicus* have been observed signalling to clients with their white antennae, but compared with the related and sympatric *A. pedersoni*, *P. yucatanicus* are visited by only a fifth as many clients and they clean only 6% of clients that visit (Titus et al., 2017), so the ectoparasites consumed during cleaning are likely not a substantial portion of this species' diet. Thus, the lack of structures that increase reflectance may imply that selection on the signal function of the antennae has not been sufficient to favour the added conspicuousness that spheres or chitin layers can confer on top of chitin alone.

Signal theory predicts that if signals communicate similar messages to receivers with similar perceptual capabilities, they should be under selection to be similar across species (e.g. convergent floral syndromes: Schiestl and Johnson, 2013). In a system such as cleaners and clients, where the messages being communicated are advertisements, the particular signal form on which species converge is expected to be one that is particularly conspicuous to the intended receiver (e.g. Marler, 1957; Stankowich et al., 2011; Stevens and Ruxton, 2011). Cleaner shrimp face a particular challenge in being conspicuous to their intended receiver, because dozens of species of fish with diverse visual capabilities (e.g. Marshall et al., 2019; Schweikert et al., 2018) can all serve as clients, and the visual environment can be highly heterogeneous, varying in water type and depth (which affects light level and spectral composition) and visual background. White (i.e. spectrally neutral) signals are particularly useful compared with colourful signals in these situations because their apparent colour is less affected by ambient light level and spectral composition, the visual system of the viewer and the visual background, especially when colour constancy is accounted for. Intriguingly, convergence can occur even for non-sympatric species like those studied here (*A. pedersoni*, *L. amboinensis* and *L. debelius* do not overlap in range). These three species all presumably need to send similar messages, and all three likely experience similar pressures on signal form from receiver and environment given that all service reef fish and live in the similar optical environments of coral reefs. Thus, convergence in this system may be at a global level, similar to the convergence in signals that advertise cleaner status that has occurred between cleaner wrasses (Labridae) and cleaner gobies (Gobiidae) across the Caribbean and Indo-Pacific (Cheney et al., 2009; Côté, 2000). The data presented here are consistent with the hypothesis that cleaner shrimp are under selection to increase the visibility of their signals in a way that is effective for a range of visual systems across diverse visual environments.

## Supplementary Material

10.1242/jexbio.248064_sup1 Supplementary information
