# Supplementary material for: Nanoscale ultrastructures increase the visual conspicuousness of signalling traits in obligate cleaner shrimps
Source: J Exp Biol. 2024 Aug 29;227(16):jeb248064. doi: 10.1242/jeb.248064 (PMC11418175; doi:10.1242/jeb.248064)
Supplement: Supplementary information [file jexbio-227-248064-s1.pdf]

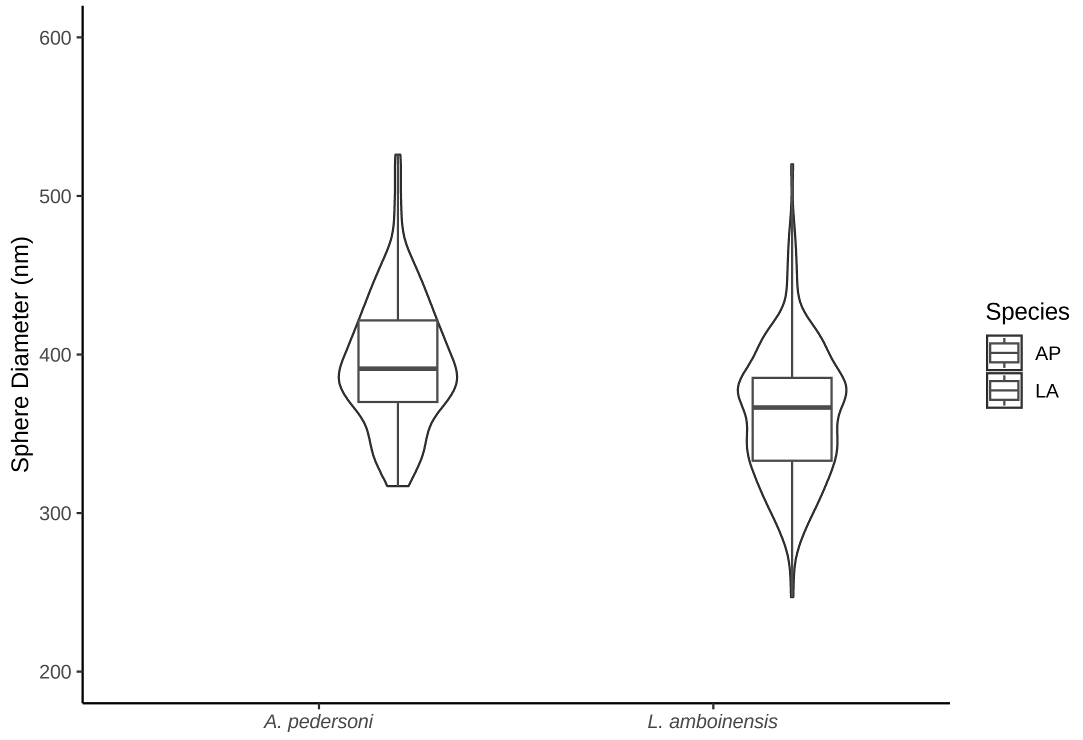

Fig. S1. Nanosphere diameter. Measurements of the nanospheres found in the antennae in both *Ancylomenes pedersoni* and *Lysmata amboinensis* show that both species generally have nanospheres between 300-450nm. The distribution of sizes, however, skews larger in *A. pedersoni*, but given that measurements came from only 2 individuals per species, we lack power for statistical tests of sphere difference between species.
